# Impact of menopause on relapse rate and disability level in patients with multiple sclerosis (MS): a systematic review and meta-analysis

**DOI:** 10.1186/s12883-023-03332-1

**Published:** 2023-09-04

**Authors:** Zahra Shahraki, Mohsen Rastkar, Elnaz Rastkar, Mehdi Mohammadifar, Aida Mohamadi, Mahsa Ghajarzadeh

**Affiliations:** 1 Zabol university of medical sciences, Zabol, Iran; 2 Student’s Scientific research center, Tehran University of Medical Sciences, Tehran, Iran; 3 Faculty of Medicine, Tabriz University of Medical Science, Tabriz, Iran; 4 Multiple Sclerosis Research Group (MSRG), Universal Scientific Education and Research Network (USERN), Tehran University of Medical Sciences, Tehran, Iran; 5 Department of Neurology, Johns Hopkins University, Baltimore, MD USA

**Keywords:** Multiple sclerosis, Menopause, Systematic review

## Abstract

**Background:**

Menopause is a physiologic phase in women’s lives. Findings regarding multiple sclerosis (MS) course through menopause are diverse. So, we designed this systematic review and meta-analysis to estimate the impact of menopause on relapse rate, and disability status in women with MS.

**Methods:**

PubMed, Scopus, EMBASE, Web of Science, and google scholar were systematically searched by two independent researchers on January 1st, 2023. They also evaluated conference abstracts, and references of the included studies. In addition, data regarding the total number of participants, name of the first author of the publication, publication year, country of origin, disease duration, disease type, annual relapse rate, and Expanded Disability Status Scale (EDSS) before and after menopause were recorded.

**Results:**

A literature search revealed 1024 records. Twenty-one full texts were evaluated, and finally, four studies were included for meta-analysis. Mean ARR before menopause ranged between 0.21 and 0.37, and after menopause ranged between 0.13 and 0.08. The SMD of mean ARR ranged between − 1.04, and − 0.29, while the pooled SMD was estimated as -0.52(95% CI: -0.88, -0.15) (I^2^ = 73.6%, P = 0.02). The mean EDSS before menopause ranged between 1.5 and 2, and after menopause ranged between 2 and 3.1. The SMD of EDSS ranged between 0.46, and 0.71. The pooled SMD of EDSS change (after menopause-before menopause) estimated as 0.56(95% CI: 0.38, 0.73)(I^2^ = 0, P = 0.4).

**Conclusion:**

The result of this systematic review and meta-analysis show that menopause can be associated with relapse rate reduction, unlike increase in disease-related disability in women with MS.

**Supplementary Information:**

The online version contains supplementary material available at 10.1186/s12883-023-03332-1.

## Introduction

Multiple sclerosis (MS) is an inflammatory, neurodegenerative disease of the central nervous system (CNS), affecting women more than men, leading to a wide range of disability and quality of life impairment [1–4]. Different factors such as genetics, sunlight exposure (vitamin D level), smoking, and Epstein-Barr virus infection are among potential risk factors of developing MS [5, 6].

Predominance of autoimmune diseases in women is clear, which is estimated 3:1 for MS [7, 8]. Gender differences about age at disease onset, progression, and inflammation are observed, and women show slower disability accumulation, delayed reach to disability milestones, and faster recovery after relapses [9–11]. On the other hand, it has been shown that before puberty, males and females have the same odds of developing MS, while after puberty, females are predominately affected [12–14].

Strong activation of T-cells, more cytokine genes expression, and increased level of immunoglobulins highlight the role of sex hormones, specially estrogen, progesterone, and testosterone, as mediators of sex differences in autoimmune diseases such as MS [15, 16].In MS, the disease activity is suppressed during pregnancy (especially during the third trimester) due to significantly increased levels of estrogen and progesterone concentrations [17]. After delivery, a rebound of CNS inflammation is more common, and women experience relapses almost three times higher than pre-pregnancy period [17, 18].

A recent systematic review and meta-analysis showed that the risk of MS decreases by increasing menarche age (12% decrease of odds of developing MS by one year increase of menarche age) [19]. Earlier age at menarche results in oestrogen balance upset, and predisposes to MS [20].

Menopause, which is characterized by long-lasting termination of ovarian follicular activity, leads to blood level decrease of oestrogen [21], which may result in disability progression, and decreased inflammatory activity in women with MS [22].

Worsening MS-related disability after menopause may suggest that deprivation of gonadal steroid is linked to neurodegeneration, which is supported findings of a previous study, showing that anti-Mullerian hormone (AMH) level (indicating ovarian age) was negatively associated with higher levels of gray matter loss, and disability [23].

Findings regarding MS course through menopause are diverse. So, we designed this systematic review, and meta-analysis to estimate impact of menopause on relapse rate, and disability status.

## Methods

We followed referred Reporting Items for Systematic Reviews and Meta-Analyses (PRISMA) 2020 [24].

### Eligibility criteria

#### The inclusion criteria were

retrospective or prospective cohort studies.

#### The exclusion criteria were

Letters to the editor, case-control, case reports, and cross-sectional studies.

#### Information sources

PubMed, Scopus, EMBASE, Web of Science, Google scholar were systematically searched by two independent researchers on January 1st, 2023. They also evaluated conference abstracts and references of the references.

#### Search strategy

The MeSH terms were.

((((((((((((((((((((((Menopause[MeSH Terms]) OR (Menopause, Premature[MeSH Terms])) OR (Postmenopause[MeSH Terms])) OR (Premenopause[MeSH Terms])) OR (Menopaus*[Text Word])) OR (Menopause, Premature[Text Word])) OR (Premature Menopause[Text Word])) OR (Postmenopaus*[Text Word])) OR (Postmenopausal Period[Text Word])) OR (Period, Postmenopausal[Text Word])) OR (Post-Menopaus*[Text Word])) OR (Post Menopaus*[Text Word])) OR (Post-menopausal Period[Text Word])) OR (Period, Post-menopausal[Text Word])) OR (Post menopausal Period[Text Word])) OR (Premenopaus*[Text Word])) OR (Premenopausal Period[Text Word])) OR (Pre-Menopaus*[Text Word])) OR (Pre menopaus*[Text Word])) OR (Pre-menopausal Period[Text Word])) OR (Pre menopausal Period[Text Word])) OR (Period, Pre-menopausal[Text Word])) AND ((((((((Multiple Sclerosis[MeSH Terms]) OR (Multiple Sclerosis[Text Word])) OR (Sclerosis, Multiple[Text Word])) OR (Sclerosis, Disseminated[Text Word])) OR (Disseminated Sclerosis[Text Word])) OR (Acute Fulminating Multiple Sclerosis[Text Word])) OR (Multiple Sclerosis, Acute Fulminating[Text Word]))).

#### Selection process, and data collection

After obtaining all retrieved studies by two independent researchers, all results were imported to the Endnote, and duplicates were deleted. Then, Titles and abstracts were screened by two researchers, and full texts of eligible studies were evaluated.

If they disagreed regarding including a study, the third party helped them. Extracted data were entered the Excel sheet by each one and checked by the third one.

#### Data items

Data regarding the total number of participants, first author, publication year, country of origin, disease duration, disease type, annual relapse rate, and Expanded Disability Status Scale (EDSS) before and after menopause were recorded.

#### Study risk of bias assessment

We assessed the risk of potential bias using the NEWCASTLE - OTTAWA QUALITY ASSESSMENT SCALE [25].

#### Effect measures

Standardized mean difference (SMD) was calculated as the effect size for annual relapse rate and EDSS. Otero-Romero et al. reported median and Inter quartile range (IQR) for EDSS, so we considered the mean as the same as median, and calculated the standard deviation from IQR (IQR/1.35).

#### Synthesis methods

All statistical analyses were performed using STATA (Version 14.0; Stata Corp LP, College Station, TX, USA).

To determine heterogeneity, Inconsistency (I^2^) was calculated.

We used fixed effects model or random-effects model for meta-analysis if the heterogeneity between study results (I^2^) was less than 50% or more than 50%.

#### Certainty assessment

For each summary estimate, we reported the pooled estimate as well as 95% CI to show certainty.

## Results

**Fig. 1:**
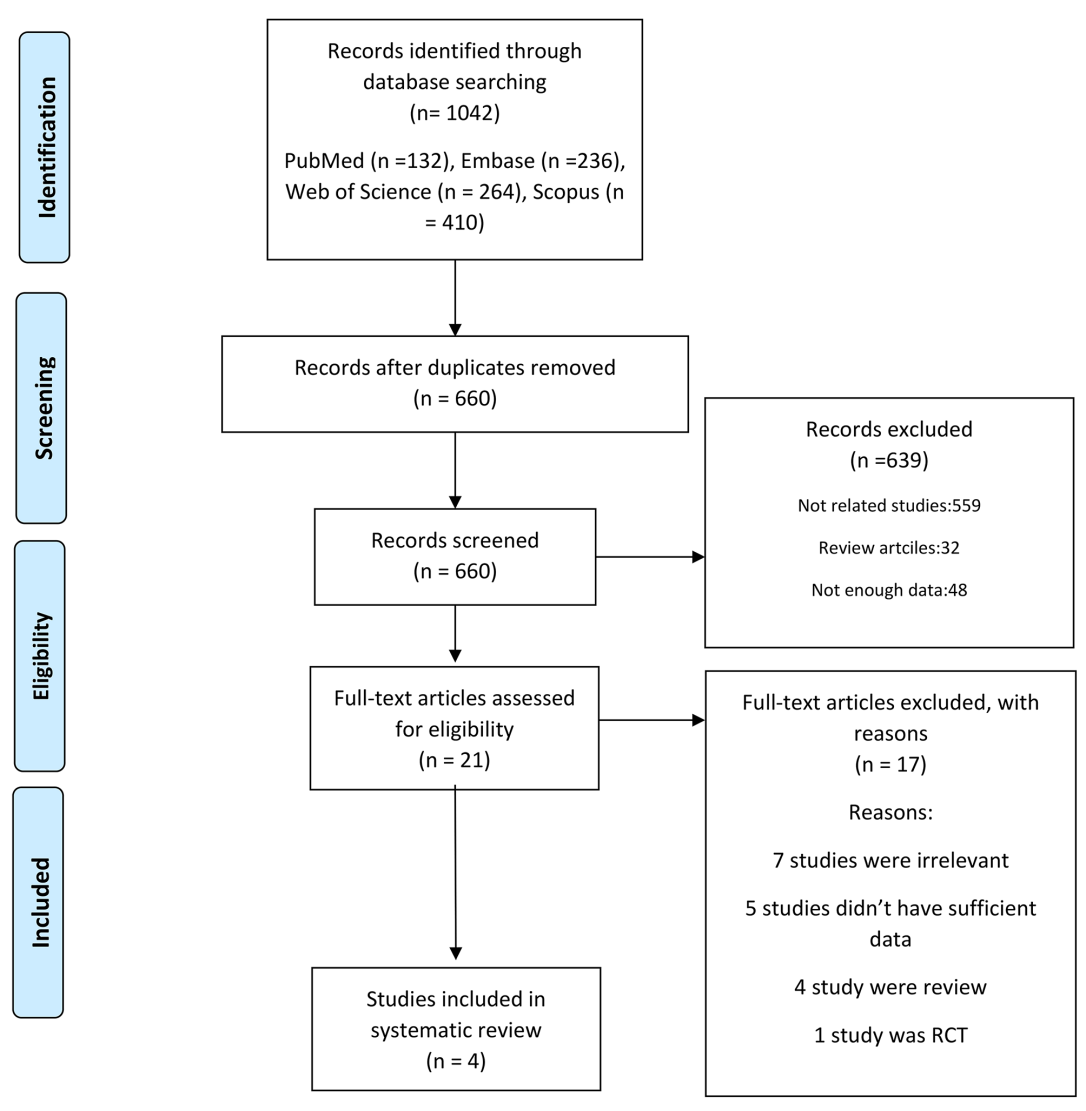
the flow chart of studies inclusion

A literature search revealed 1024 records. After deleting duplicates, we had 660 records. Twenty-one full texts were evaluated. Finally, four studies remained for meta-analysis (Fig. 1).

The included studies were published between 2017 and 2020. Two were conducted in Italy, one in Portugal, and one in Spain. The number of enrolled patients ranged between 37 and 148, and mean age ranged between 47 and 50 years. One study was a conference abstract (Baroncini et al. 2017) (Table 1).

**Table 1:** Data extracted from studies

Study ID	Author name	Year	Country	Study design	Number of participants	Age of menopause	Disease duration	Disease type	Follow up time	Annual relapse rate		EDSS		NOS score
										Before	After	Before	After	
1	Baroncini et al [21].	2019	Italy	Retrospective Cohort	148	50.3 ± 3.8	-	-	3.7 ± 0.6 years before and 3.5 ± 0.7 years after menopause onset	0.21 ± 0.31	0.13 ± 0.24	2 ± 1.2	2.6 ± 1.4	7/9
2	Baroncini et al [26].	2017	Italy	Retrospective Cohort	84	50.2 ± 3.2	14.6 ± 7.8	-	2–4 years pre and post- final menstrual period	0.23 ± 0.28	0.13 ± 0.23			-
14	Ladeira et al [22].	2018	Portugal	Retrospective Cohort	37	49.8 ± 4.06	14.0 ± 8.64	28 RR, 2 PP, 7 SP	At least 1 year before and after menopause	0.37 ± 0.35	0.08 ± 0.18	2 ± 1.06	3.1 ± 1.93	7/9
16	Otero-Romero [27]	2020	Spain	Prospective Cohort	74	47.2 ± 5.6	-	74 CIS	Median follow-up from onset of 13.3 years (SD 4.4) and a median postmenopause observation pe-riod of 6.03 years (SD 4.7)			Median (IQR) 1.5(1-1.5)	Median (IQR) 2(1.1–2.5)	6/9

**Fig. 2:**
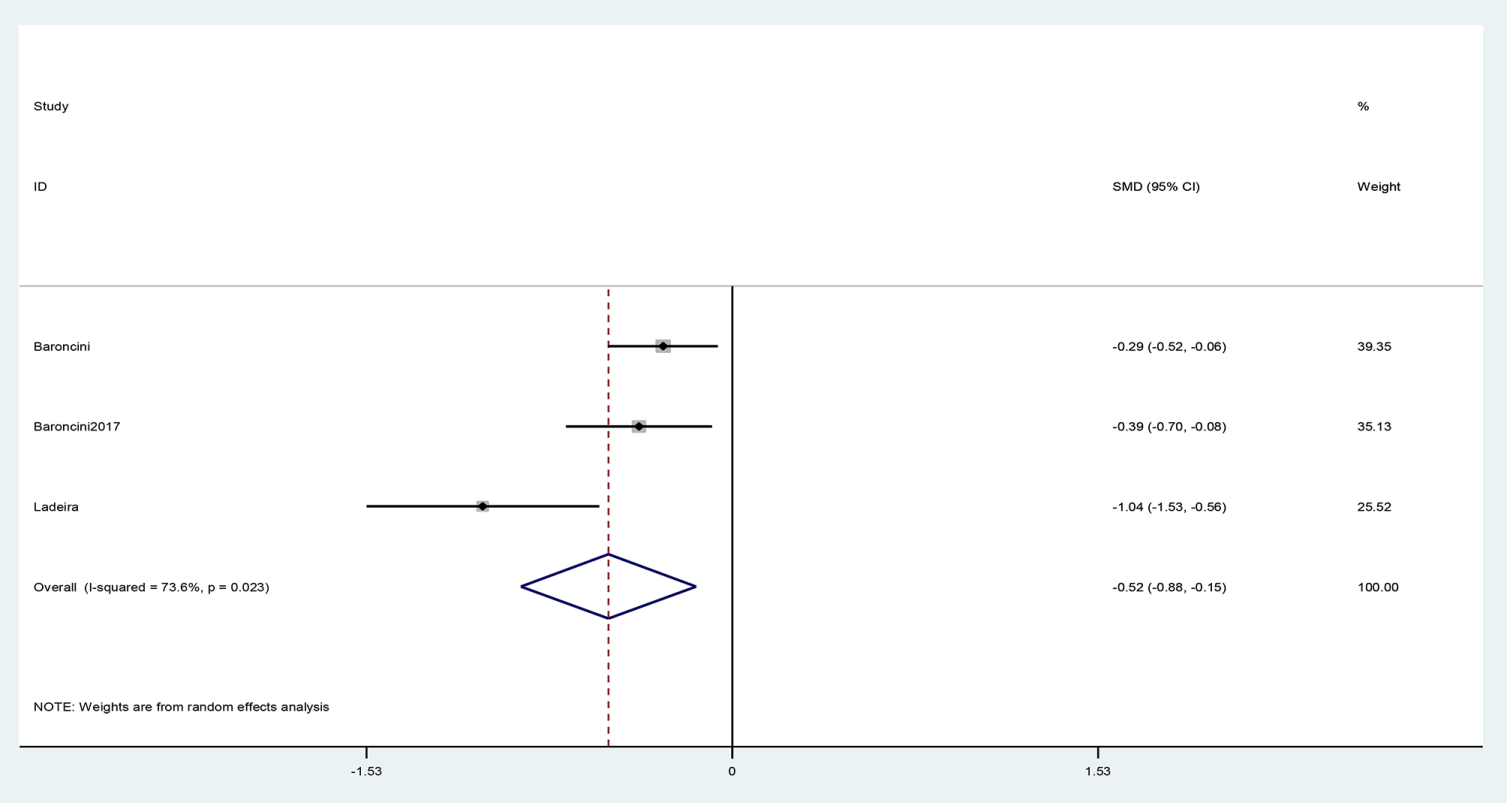
The pooled Standardized mean difference (SMD) of annual relapse rate analysis (ARR) (after menopause -before menopause)

The SMD of mean ARR ranged between − 1.04, and − 0.29, while the pooled SMD was estimated as -0.52(95% CI: -0.88, -0.15) (I^2^ = 73.6%, P = 0.02) (Fig. 2).

Mean EDSS before menopause ranged between 1.5 and 2, and after menopause ranged between 2 and 3.1. the SMD of EDSS ranged between 0.46, and 0.71.

**Fig. 3:**
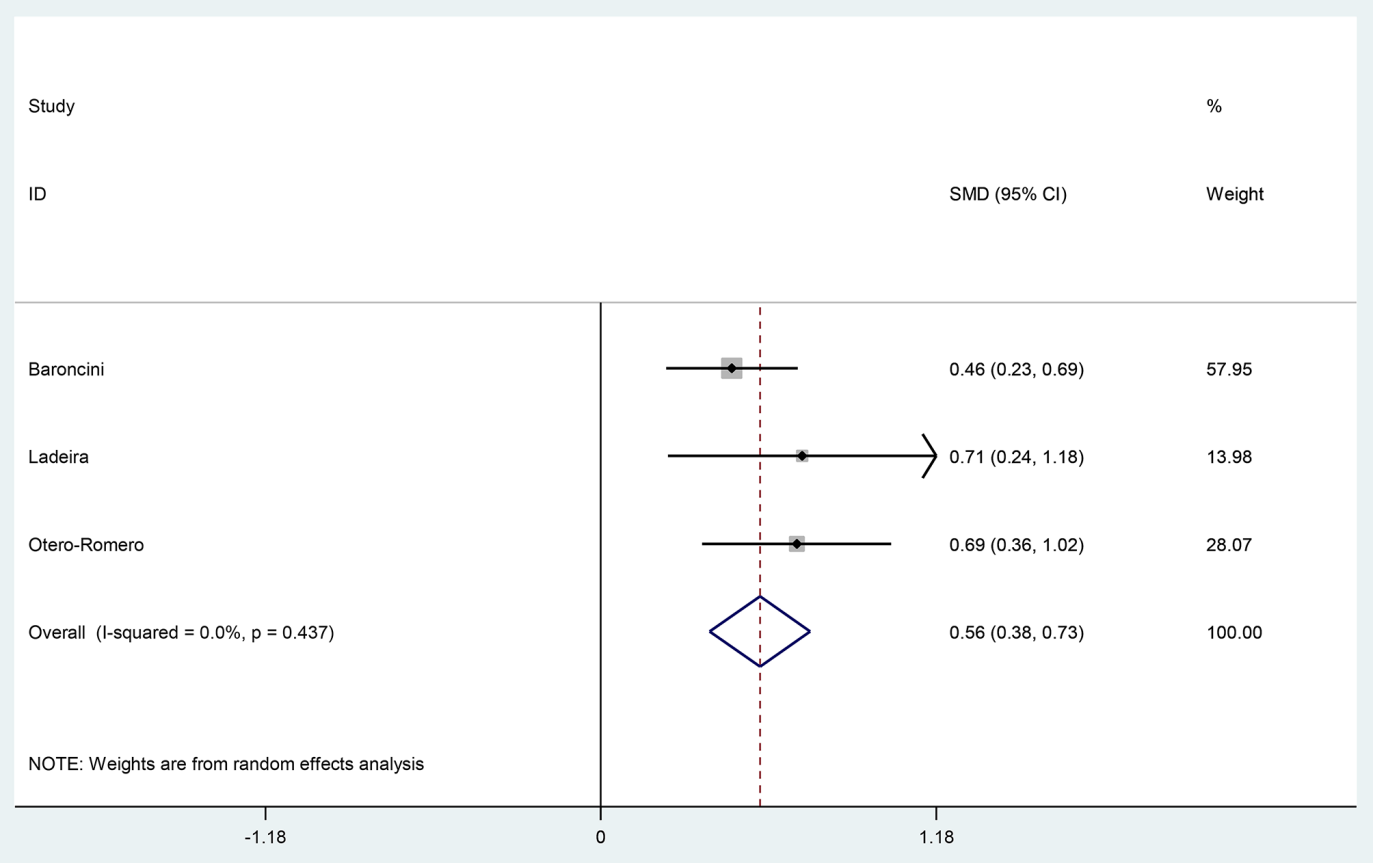
The pooled Standardized mean difference (SMD) of Expanded Disability Status Scale (EDSS) (after-before study)

The pooled SMD of EDSS change estimated as 0.56(95% CI: 0.38, 0.73) (I^2^ = 0, P = 0.4)(Fig. 3).

## Discussion

Menopause is a health issue that is not well considered in women with MS. Women with MS may face challenges regarding managing menopause as well as controlling their disease-related symptoms as the effects of menopause on relapse rate, and MS-related disability remain understudied.

Persistent decrease of oestrogen level after menopause may lead to MS course alteration. Immunosenescence as the consequence of sex steroid production reduction after menopause, includes lymphocyte telomere lengths decrease, and immune response alteration (cellular, and humoral) [28, 29].

To our knowledge, this is the first comprehensive and systematic review, including meta-analysis regarding impact of menopause on MS-related relapses and disability.

The previous systematic review in this field, only included two studies in meta-analysis, and found that menopause does not affect relapse rate [30].

Our results show that after menopause, women with MS experience less disease-related relapses compared to reproductive time, but we could not conclude that its only due to menopause as relapse rate which indicates inflammation in MS is associated negatively with age, and relapse rate reduction could be detected by advanced age [31].

In a retrospective, observational study, Baroncini et al. included 148 women with MS (3.5 years before the final menstrual period, and after the final menstrual period). They found that ARR significantly decreased after menopause (38% reduction, p = 0.005) [21].

Their findings were along with the findings of Ladeira et al. who enrolled thirty- seven post-menopausal women with mean age of 49.8 years. During five years follow up, they investigated significant reduction of ARR (0.08 ± 0.18 post-menopause vs. 0.37 ± 0.35 pre-menopause) [22].

Alternatively, we found that disease-related disability which was measured by EDSS, increased significantly. This indicates the negative effect of menopause on disease course in women, but it should be noted that disability increases significantly by increasing age in patients with MS, and age is a significant predictor of disability progression in MS [32].

In Baroncini et al. study, the mean EDSS increased from 2 to 2.6, and in Ladeira et al. study, the mean EDSS changed from 2 to 3.1after menopause [21, 22].

In a longitudinal study, Otero-Romero et al., evaluated EDSS in women with clinically isolated syndrome (CIS) or MS. Among 764 eligible women, 94 enrolled in the final analysis. They found that annual increase in EDSS was significantly higher in menopausal women compared to non-menopausal women (0.049 vs. 0.019) (p value 0.02) [27].

Sex hormones contribute in developing MS, and explain the sex inequality for MS incidence [33]. Estrogens are sex steroid hormones, including estrone (E1), 17β-estradiol (E2), and estriol (E3). In pre-menopausal women, E2 is dominant while during pregnancy E2, and E3 are elevated.

Estrogen affects B cell’s maturation, differentiation, and survival [34–36], and we know that production of anti-myelin antibodies, presenting antigens, and producing cytokines are done by B cells [37, 38].

Higher levels of oesterogen also lead to T helper 1 to T helper 2 shift in the immune system which cause changes in immune responses [39]. The explanation for why the relapse rate decreases after menopause could be due to the reduction of CD4 T and B lymphocytes (immunosenescence),both in number and function, due to aging and the nature of the menopause [40, 41].

After menopause reduced numbers of B cells, and T cells, and increase in pro-inflammatory cytokines are obvious which could be due to age-related immunosenescence [33]. Increased level of IL-6, and tumor necrosis factor–α (TNF- α), as well as oxidative stress after menopause indicate low grade inflammation [29, 41]. It has been shown that hormone replacement therapy after menopause is associated with decreased level of interleukin-6, and natural killer cell activity [42, 43].

On the other hand, the increase in disease-related disability after menopause could be due to decreased oestrogen level, and lack of its neuroprotective effects [21]. Both human, and animal studies showed that oestrogen has neuroprotective effects, leading to better neural survival [44–47]. It has been shown that oestrogen treatment is associated with glutamate-induced apoptosis decrease, reduction of cytotoxicity of oligodendrocytes, and oligodendrocyte process formation acceleration [48–50].

It is evident that the anti-inflammatory role of oestrogens after menopause decreases, which leads to aggressive, inflammatory damage of axons plus myelin sheets, as well as disability progression [51].

This systematic review has some strengths. First, it includes more studies as well as meta-analysis. Second, we analyzed both relapse rate, and disability status after menopause.

We had some limitations too. First, all studies had no data regarding type of disease and duration of the disease. Second, only one study had prospective design. All studies did not provide information regarding DMT. In meta-analysis of a smaller cohort of studies, the I^2^ is not a good representative of heterogeneity. EDSS data or ARR data were not provided by all studies.

## Conclusion

The result of this systematic review and meta-analysis show that menopause can be associated with relapse rate reduction, unlike increase in disease-related disability in women with MS.

### Electronic supplementary material

Below is the link to the electronic supplementary material.

Supplementary Material 1

## Data Availability

All data generated or analyzed during this study are included in this published article.
